# Dual Plating and Dorsal Bridge Plating for Comminuted Distal Radius Fractures: A Systematic Review

**DOI:** 10.3390/jcm15155991

**Published:** 2026-08-01

**Authors:** Sami Chergui, Abdullah AlOmar, Abdurahman Addweesh, Muan AlMoajil, Rudolf Reindl

**Affiliations:** 1 Department of Orthopedic Surgery, McGill University, Montreal, QC H3H 1V8, Canada; 2 Division of Orthopedic Surgery, Prince Sultan Medical City, Riyadh 11159, Saudi Arabia; 3 Faculty of Medicine, King Saud University, Riyadh 11451, Saudi Arabia

**Keywords:** distal radius fracture, bridge plating, dual plating, orthopedic trauma

## Abstract

**Background/Objectives**: Complex AO type C distal radius fractures remain amongst the most difficult injuries to treat, often requiring advanced fixation techniques. This systematic review focuses on two commonly used strategies for complex injuries: combined dorsal and volar plating and dorsal bridge plating. The goal is to examine reported functional and radiographic outcomes as well as complications of both techniques. **Methods**: A search was conducted across Embase, Medline, and Web of Science following PRISMA guidelines. Studies were included if they reported clinical or radiographic outcomes in adult patients with complex distal radius fractures treated using either combined dorsal and volar plating or dorsal bridge plating. **Results**: Sixteen studies were included, encompassing a total of 629 distal radius fractures, 419 treated with combined plating and 210 with bridge plating. Both techniques were associated with improved wrist function and satisfactory radiographic alignment. The weighted mean Disabilities of the Arm, Shoulder, and Hand (DASH) score was 12.07 for combined plating and 25.20 for bridge plating. Radiographically, both techniques restored acceptable volar tilt, with a weighted mean of 5.97° for combined plating and 3.32° for bridge plating. Overall complication rates were low, with reported infection rates of 2.86% for both techniques. Revision rates for complications were 4.30% and 5.71% in dual and bridge plating, respectively. Tendon injuries occurred in 1.19% of patients after combined plating and 1.43% after spanning fixation. **Conclusions**: This review highlights the promising results of both combined plating and dorsal bridge plating in the treatment of complex distal radius fractures. As surgeons continue to navigate these challenging injuries, further comparative studies are required to determine the optimal management strategy.

## 1. Introduction

Complex distal radius fractures with articular involvement and metaphyseal comminution continue to challenge orthopedic surgeons. High-energy injuries, often accompanied by ligamentous disruption and extensive comminution, create a setting where traditional fixation methods may not offer sufficient stability [1,2]. Historically, external fixation (EF) was used to treat high-energy fractures. However, its limitations (such as pin track infections, suboptimal stabilization in severely comminuted fractures and hindrance to early mobilization) have driven the search for better alternatives [1,3].

Over the past decades, the management of these fractures has shifted toward plate fixation. The advent of volar locking plates (VLPs) marked a significant advancement by allowing early range of motion (ROM) and avoiding complications associated with EFs [4,5]. Despite these advantages, VLPs can be insufficient in cases with dorsal comminution or where unstable dorsal fragments are present [6,7,8]. As a response, surgeons have adopted hybrid techniques (including combined volar and dorsal plating) to better reconstruct the complex geometry of the distal radius [5,6,9,10].

Spanning or bridge plating was first described by Burke and Singer in 1998 [11]. This technique temporarily bridges the radiocarpal joint to act as an internal fixator, stabilizing the fracture by spanning the dorsal comminution. Spanning plates have shown particular promise in elderly or polytrauma patients [9]. Similarly, the combined use of volar and dorsal plates has emerged as a reliable method for addressing complex intra-articular distal radius fractures. This dual approach allows surgeons to sequentially stabilize both volar and dorsal fragments, particularly when single plating techniques fall short in managing central impaction or dorso-ulnar instability. This method provides multidirectional fixation, improving articular congruity and mechanical stability [12].

Collectively, the current literature underscores the importance of tailoring the fixation strategy to the specific fracture pattern. While VLPs have become the mainstay of treatment, the evolving evidence on spanning and dual plating suggests that these methods may offer particular advantages in complex distal radius fractures. To our knowledge, there are currently no systematic reviews focusing on both combined plating and bridge plating techniques in complex (AO type C) distal radius injuries [13]. Thus, this systematic review aims to evaluate clinical, functional, and radiographic outcomes of these dual and bridge plating techniques in the context of AO type C distal radius fractures.

## 2. Materials and Methods

The systematic review adhered to the guidelines outlined by the PRISMA (Preferred Reporting Items for Systematic reviews and Meta-Analyses) standards for data reporting. The PRISMA checklist can be found in Table S1. The protocol was registered with the International Prospective Register of Systematic Reviews (PROSPERO) [CRD420261413203].

The search strategy was used within Embase, Medline, and Web of Science. Screening was independently performed by two reviewers. The search strategy can be found in Appendix A. Discrepancies were resolved by a third reviewer. Inclusion criteria were as follows: prospective or retrospective clinical research and published from 1 January 2004 to 1 March 2025. The studies must have examined radiological, clinical or functional outcomes for skeletally mature individuals with complex distal radius fractures treated with either dual plating or temporary spanning plate fixation. Exclusion criteria comprised non-clinical studies, review articles, studies on non-human subjects and studies not published in English.

The quality of each selected article was evaluated by two authors using the “Methodological Index for Non-Randomized Studies (MINORS)” tool, as developed and validated by Slim et al. [14]. This tool allows a maximum score of 16 for non-comparative studies, with higher scores reflecting superior study quality. In instances of scoring conflicts, average scores were determined. The Grading of Recommendations Assessment, Development, and Evaluation (GRADE) tool was also used to evaluate the strength of recommendations and outcomes. It appraises the certainty of recommendations based on study design, risk of bias, effect size, outcome consistency, plausible effect and precision [15,16].

Extracted data of interest included study type, patient demographics, fixation technique, follow-up duration, clinical outcomes, radiological results, and complications. Given the heterogeneity of the included studies and limited data, a meta-analysis was not performed. Weighted means were calculated based on the number of wrists within each study to synthesize demographic data, and outcomes were compiled by calculating total proportions. The data were synthesized for descriptive purposes and without the intent to perform any comparative analysis between the treatment methods. Authors were contacted to obtain missing data. If data could not be obtained, then weighted means were performed with the remaining studies, and the study with missing data was excluded from that particular analysis.

## 3. Results

**Figure 1:**
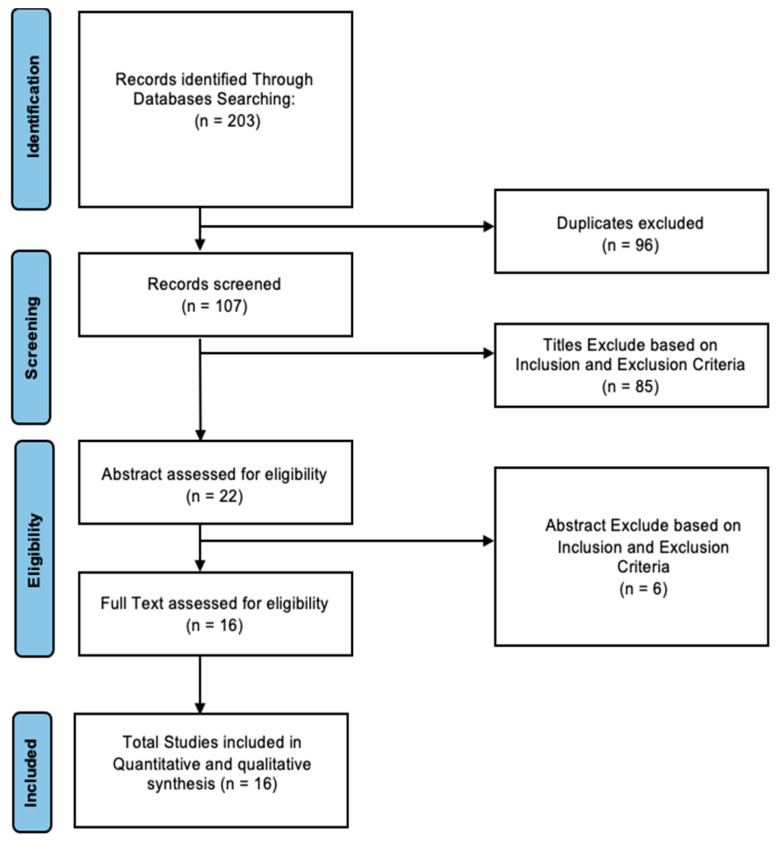
PRISMA Flowchart of Screened Studies.

The search strategy yielded 203 articles. After removing 96 duplicates, 107 unique articles remained. Title screening excluded 85 articles. Abstract review led to the exclusion of six additional articles based on the inclusion criteria. Sixteen full-text articles were reviewed in detail. No further articles were included from reference lists (Figure 1).

This review included 15 retrospective case series and one randomized controlled trial. The average critical appraisal score was 10.02 ± 1.62 on a 16-point scale. Mean scores of both reviewers were 10.08 ± 1.62 and 9.95 ± 1.66, respectively. Common deductions were due to unblinded data collection, retrospective design and lack of *p*-values or confidence intervals. Interobserver agreement was 83% with a Cohen’s kappa of 0.86, indicating excellent reliability. The MINORS tool was not applied to the randomized controlled trial (RCT), as it was not designed for that type of study.

The review encompassed 629 wrists. Sex was reported in 13 of 16 studies, showing 36.05% male patients (212/588). The weighted mean age was 57.05 ± 7.51 years, with a follow-up duration of 25.15 ± 27.01 months.

Eight articles focused on combined plating (419 wrists), with a mean age of 57.27 ± 4.84 years and 32.95 ± 29.72 months of follow-up. Fracture types were clearly identified in 341 wrists. There were 34 AO type C1, 104 type C2, and 203 type C3 injuries. Bone grafting was used in 5/8 studies; comorbidities were not reported.

Bridge plating was described in eight articles (210 wrists). Mean age was 56.62 ± 11.05 years. Mean follow-up was 9.59 ± 7.72 months. Fracture classification was available for 177 wrists: six type C1, 19 type C2 and 152 type C3. Bone grafting was reported in 2/8 studies. Comorbidities included osteoporosis (24/60), smoking (24/60), and diabetes (8/60) across two studies (Table 1).

In combined plating studies, functional outcomes were measured using the Disabilities of the Arm, Shoulder, and Hand (DASH/QuickDASH) score in 7/8 studies. These articles yielded a mean score of 12.07 ± 5.69. Patient-Rated Wrist Evaluation (PRWE) scores averaged 12.80 ± 6.75. ROM showed mean extension of 53.35 ± 4.49° and flexion of 52.80 ± 5.13°. Grip strength averaged 84.45 ± 8.64% of the contralateral side. Radiographic outcomes included a mean Batra score of 88.0 ± 0.0, average volar tilt of 5.97 ± 3.52°, radial height of 10.99 ± 1.39 mm, and articular step-off of 0.87 ± 0.14 mm (Table 2).

Bridge plating studies reported DASH/QuickDASH in 5/8 studies (mean 25.20 ± 6.07). Only one study reported a PRWE score, which was 14.00. ROM averaged 50.89 ± 4.83° of extension and 46.85 ± 2.66° of flexion. Grip strength was 86% of the contralateral side in one study. Radiographic values included volar tilt (mean 3.32 ± 1.84°) and radial height (mean 10.24 ± 0.74 mm). Articular step-off (average 0.88 ± 0.53 mm) was reported in two studies (Table 2). Comparisons could not be made directly between both techniques due to the quality and heterogeneity of the available data.

In the combined plating group, superficial and deep infections occurred in 12/419 patients (2.86%), with one-third of these cases requiring operative debridement. Implant removal occurred in 148/419 patients (35.32%) for irritating hardware. Revision surgeries, other than hardware removals, were required in 18/419 wrists (4.30%). Excluding hardware removals and washouts of infections, corrective osteotomies and management of tendon injuries were the most common revision procedures. Tendon injuries were reported in 5 (1.19%) patients. Three tendon ruptures required intervention. One patient suffered ruptures of the extensor pollicis longus (EPL), extensor carpi radialis longus (ECRL) and extensor carpi radialis brevis (ECRB) tendons. This was treated using extensor indicis propius (EIP) to EPL transfer as well as ECRB and ECRL repair with palmaris longus (PL) interposition grafting. Another patient experienced flexor pollicis longus (FPL) rupture, which was managed with PL interposition graft. The third patient underwent extensor digitorum communis (EDC) repair. The other two patients who did not undergo repair had EDC and FPL injuries, respectively. Complex regional pain syndrome (CRPS) was noted in 5/341 patients (1.47%) within the studies reporting this complication.

In the bridge plating group, complications and rates of revision surgery were compiled while excluding hardware removals, as they are a planned secondary procedure when undergoing bridge plating (unless in the context of broken hardware). Implant removal was performed in all cases, but the plate had to be removed in three patients (1.43%) due to hardware failure. Revision surgery was required in 12/210 cases (5.71%). Infections occurred in six patients (2.86%) and required reoperation in one case. Flexor or extensor tenolysis due to post-operative digit stiffness composed a third of the revision operations. Tendon injuries were reported in 3/210 (1.43%) patients, including three EPL ruptures requiring EIP to EPL transfers. Amongst studies reporting rates of CRPS, 3/151 patients (1.99%) suffered from this condition post-operatively (Table 3). Comparative analysis could not be performed between both treatment options due to the quality and heterogeneity of the available data.

## 4. Discussion

Complex intra-articular distal radius fractures, particularly AO type C injuries, are technically demanding due to comminution, metaphyseal instability, and involvement of the radiocarpal and distal radioulnar joints [25]. While often associated with high-energy trauma, large-scale registry data indicate most occur in older adults following low-energy mechanisms [26]. Sagerfors et al. noted type C3 fractures still carried the highest relative rates of high-energy trauma (16.2%) and open fractures (5.8%) among C subtypes [26]. Mader and Pennig likened these injuries to tibial plafond fractures, emphasizing that subchondral bone collapse and multifragmentary joint depression pose significant challenges to anatomic reconstruction. Failure to restore key articular elements, particularly the volar lunate facet and dorsal rim, can lead to dysfunction and post-traumatic arthritis [27]. From an anatomical standpoint, Rhee et al. proposed a column-based fixation strategy addressing the radial, intermediate, and ulnar columns sequentially to restore joint congruity and prevent instability, especially in fractures involving the volar rim or dorsal ulnar corner [25].

Wadsten et al. showed that distal radius fractures with volar comminution had a 96% displacement rate, compared to just 16% in non-comminuted fractures [28]. This highlights the critical role of volar cortical integrity in maintaining stability and the need for prompt surgical intervention. Rogachefsky et al. reported that even with near-anatomic reductions in AO type C3 fractures treated with combined internal and external fixation, functional outcomes remained limited. Grip strength and flexion-extension arc reached 73% and 72%, respectively, compared to the contralateral side with post-operative articular incongruity correlating with functional scores [29]. These findings collectively emphasize that severe comminution makes obtaining and maintaining anatomic articular reduction more difficult, leading to poorer function post-operatively.

The rationale for combined volar and dorsal plating in intra-articular distal radius fractures lies in achieving three-dimensional anatomical reconstruction and stability, particularly in AO/OTA type C fractures with significant comminution. Jung et al. outlined key indications for this approach, including free-floating articular fragments, distally migrated dorsal components, or centrally impacted fragments not manageable with volar-only fixation [30]. While volar plating restores radial height and tilt, it may inadequately address dorsal instability or obscure articular defects. Dorsal fixation enables direct visualization and stabilization of key structures such as the dorso-ulnar corner and dorsal lunate facet, enhancing radiocarpal and distal radioulnar joint (DRUJ) stability. This dual approach provides greater construct rigidity and fragment-specific control of the reduction, being particularly beneficial in osteoporotic or compromised metaphyseal bone [6].

In contrast, dorsal bridge plating (DBP) was introduced by Burke and Singer in 1998 to manage highly comminuted distal radius fractures when conventional fixation fails or when it is deemed that direct reduction and fixation of articular fragments is not feasible [11]. The technique spans the radiocarpal joint, using ligamentotaxis to indirectly reduce the fracture and allowing early mobilization of adjacent joints. It is particularly useful in polytraumatized or elderly patients with poor bone quality, or in cases with severe comminution and compromised soft tissues, such as open fractures [20,24]. DBP uses remote, minimally invasive incisions, preserving soft tissue integrity. Traditionally, these severely comminuted fractures would be treated via external fixator. However, data suggest that bridge plating leads to similar functional and radiological outcomes while avoiding pin-related complications [31]. Spanning plates are also particularly interesting in the context of polytraumas, as they would allow earlier weightbearing on the affected upper extremity. However, it has been suggested to use 3.5 mm plates instead of 2.4 mm plates in this context [32]. This in turn could affect post-operative extensor tendon attrition given the increased hardware size.

Both techniques are associated with different drawbacks. Dual plating requires two incisions and extensive dissection, which may increase operative time and soft-tissue complications [30]. Dorsal implants, in particular, are associated with extensor tendon irritation or rupture, most commonly involving the EPL tendon [6,7]. Gruenberger et al. noted increased operative duration and hardware-related tendon injuries with dorsal plates [10]. Similarly, Lundqvist et al. highlighted the invasiveness of this approach towards the constrained dorsal compartments [18]. Hadzhinikolova et al. confirmed the biomechanical benefits in dorsal lunate facet comminution but noted that additional dissection may not be warranted in less complex patterns [8]. Furthermore, implant removal is common due to dorsal compartment tendon irritation. Sagerfors et al. reported a 28% removal rate [5]. Finally, intraoperative judgment about the need for dorsal fixation can be challenging and may lead to overtreatment [30].

DBP also has notable limitations. Since the construct spans the wrist, a period of immobilization is needed, delaying range of motion exercises and restricting wrist function [4,20]. A planned second procedure is required for plate removal, adding anesthetic and logistical burdens that can be challenging in frail patients or in low-resource centers [20,33]. The review by Drummond et al. demonstrated that authors would remove the plate at a mean of 17.3 weeks after the initial surgery [33]. Surgically, the technique typically involves three incisions, possible EPL tendon transposition and meticulous dissection around dorsal compartments. This can raise the risk of complications like tendon adhesions and digital stiffness, especially with overdistracted constructs or suboptimal post-operative physiotherapy [34]. Wrist stiffness is a constant concern. Gruenberger et al. found most ROM gains occurred within 4 weeks after plate removal, with limited improvement afterwards [10]. Drummond et al. reported digital stiffness in 18.2% of DBP cases, but outcomes improved when tenolysis was performed during plate removal. Complication rates were higher in patients whose plates remained over 16 weeks (20.8%) compared to earlier removal (8.5%), suggesting that timely hardware removal may reduce postoperative stiffness and other complications [33]. In this current review, digital stiffness requiring tenolysis was present in 1.90% of bridge plating patients. Studies have also shown that bridge plating fixation to the 2nd metacarpal yielded more favorable ROM and grip strength compared to fixation to the 3rd metacarpal [35,36].

Pooled analysis of combined dorsal-volar plating showed favorable functional outcomes, with a weighted mean DASH/QuickDASH score of 12.07 ± 5.69 and PRWE of 12.80 ± 6.75. Mean wrist extension and flexion were 53.35 ± 4.49° and 52.80 ± 5.13°, respectively. Grip strength averaged 84.45 ± 8.64% of the contralateral side. In the dorsal bridge plating group, DASH/QuickDASH scores were 25.20 ± 6.07, and PRWE (reported in one study) was 14.00. Mean extension and flexion were 50.89 ± 4.83° and 46.85 ± 2.66°, while grip strength was 86%. When reviewing outcomes of VLP fixation, the literature reported DASH scores ranging from 5.4 to 10.5 and PRWE from 6.1 to 12.7. Wrist extension and flexion ranged between 59–64° and 57–64°, respectively [37,38]. Grip strength typically ranged from 79 to 88%. External fixation (EF) cohorts showed broader variability: DASH scores between 20 to 23, PRWE from 6.6 to 12.7, and grip strength between 76% and 90% [38,39]. Despite not being able to perform direct comparisons between dual and bridge plating, both techniques offer functional benefits in the context of complex distal radius fractures.

Radiographically, dorsal-volar plating yielded a weighted mean volar tilt of 5.97 ± 3.52°, radial height of 10.99 ± 1.39 mm, and articular step-off of 0.87 ± 0.14 mm. DBP cases showed volar tilt of 3.32 ± 1.84°, radial height 10.24 ± 0.74 mm, and step-off 0.88 ± 0.53 mm. Similarly, VLP studies reported volar tilts of 3–8°, radial heights of 10–11 mm, and step-offs under 1 mm [37,38]. EF article results were more variable, with some cases reporting displacement or step-offs > 2 mm [39]. Nonetheless, all techniques generally achieved satisfactory alignment.

Complications were low across all plating groups. Infections occurred in 12/419 (2.86%) and 6/210 (2.86%) of patients undergoing dual and bridge plating, respectively. Revision surgery, excluding hardware removals, was required in 4.30% of cases with combined plating and in 5.71% of patients with spanning plates. It is also important to note that the combined plating group required hardware removal in 35.32% of cases. Thus, in over a third of cases, dual plating did not confer the advantage of avoiding secondary surgery for hardware removal, which is supposed to be one of its main benefits compared to bridge plating. Tendon injuries occurred in 1.19% of dual plated wrists and 1.43% of bridge plated wrists. The most commonly injured tendon was the EPL, and EIP to EPL transfer was the most common reconstruction technique. The prevalence of extensor tendon injury can be attributed to trauma at the time of injury or at the time of surgery. However, gradual tendon attrition against the dorsal plate has been deemed as the most significant factor for these tendon injuries. Similarly, Sanchez et al. reported a rate of extensor tendon injury with dorsal radius plating of 1.29% and a rate of hardware irritation requiring removal of 75.50% of dorsal plates [40]. In contrast, VLP infection rates ranged from 0 to 3.7%, with some deep infections requiring debridement [37,38]. Flexor tendon injuries with VLP occurred in 1–1.4% of cases due to attrition against the plate [37,38]. VLPs were associated with a rate of up to 12.5% of extensor tendon injuries, especially EPL, due to prominent screws [41]. EF showed higher complication rates. One series reported pin tract infections in 9/24 patients, nerve irritation in 5/24, and loss of reduction in 4/24 cases [42]. Tendon injuries were rare in EF, but total minor and major complication rates were generally higher than with internal fixation [39,42]. From the available data, it does seem that both dual and bridge plating offer a relatively low complication profile.

This systematic review has multiple strengths. To our knowledge, this is the first systematic review consolidating data on dual and bridge plating in AO type C fractures specifically. Furthermore, PRISMA guidelines were followed, ensuring standardization of the article screening process. This review also provides a comprehensive overview of outcomes by describing functional as well as radiographic outcomes and complications. Consequently, this systematic review provides surgeons an exhaustive overview of dual and bridge plating to aid in clinical decisions. However, this review has limitations. Only one RCT was included, and all other studies were retrospective. Furthermore, most studies did not have a control group, potentially being exposed to selection and reporting biases. Heterogeneity in populations, fracture patterns, post-operative protocols and outcome measurements complicate direct comparisons. This heterogeneity precluded performing a meta-analysis or any comparative analysis. The heterogeneity in fracture severity led to the bridge plating group having a significantly greater proportion of AO type C3 fractures. Thus, the functional and radiographic outcomes of that cohort of patients may be attributed to greater baseline injury severity. Important confounders, such as comorbidities and rehabilitation protocols, were inconsistently reported. Additionally, the duration of bridge plating prior to hardware removal was not standardized and may have influenced both complication rates and functional outcomes. Several studies had short follow-up periods, and the mean follow-up duration in bridge plating studies was significantly shorter than in the dual plating groups. Thus, the short follow-up in the bridge plating studies may affect the reported functional outcomes and may limit the detection of late post-operative complications. Baseline radiographic parameters were not provided within the studies. Consequently, comparisons between radiographic outcomes post-operatively were restricted.

Based on the GRADE tool, the certainty of any recommendation would be classified as low, and this review cannot offer significant comparative recommendations based on the available data. The certainty of recommendations was negatively affected by the retrospective nature of almost all included studies and the lack of high-quality randomized controlled trials. Furthermore, the directness criterion of the GRADE system evaluates the ability of the available data to answer a specific question, such as comparing two treatment options. However, there is a paucity of comparative research contrasting dual plating directly with bridge plating, and no study directly addresses this question. The GRADE tool also examines the consistency and precision of results. This criterion slightly improved the certainty grading, as there was no major variability in outcomes or any significantly contradictory results between included studies. Similar findings were observed when using the MINORS criteria to evaluate the studies. The most common deductions in this scoring system were due to unblinded data collection, retrospective design and the lack of *p*-values or confidence intervals. Overall, the GRADE and MINORS tools highlight the need for prospective, standardized and randomized research. However, this review offers a comprehensive summary of the literature regarding both fixation strategies in the specific context of AO type C distal radius fractures.

## 5. Conclusions

In conclusion, this systematic review provided an overview of combined plating and dorsal bridge plating, which are now mainstay techniques in managing complex distal radius fractures. The current literature suggests both approaches can yield satisfactory functional and radiographic results in AO type C distal radius fractures. However, direct comparisons between both techniques are limited due to the lack of direct comparative studies. The decision to proceed with dual plating or bridge plating should be made on a case-by-case basis considering patient factors, fracture pattern and surgeon preference. Further prospective comparative studies are warranted to guide surgical decision-making in these challenging cases.

## Figures and Tables

**Table 1:** Patient Demographics.

Authors	Procedure	Type	Number of Patients (Wrists)	Age (Years)	Males (%)	AO Classification (Number of Patients)	Follow-Up (Months)	Bone Grafting Used
Medlock et al., 2018 [9]	Combined Plating	Retrospective case series	16 (18)	42.6	13 (72%)	C2 (15) C3 (3)	27	7 (39%) Autograft
Karlsson et al., 2020 [17]	Combined Plating	Retrospective cohort study	78 (78)	56	23 (29.5%)	C (78)	12	17 (21%) Autograft (3) Allograft (14)
Lundqvist et al., 2022 [2]	Combined Plating	Retrospective case series	97 (97)	57	30 (30.9%)	C1 (4) C2 (18) C3 (75)	84	-
Sagerfors et al., 2020 [5]	Combined Plating	Retrospective cohort study	74 (74)	62	19 (25.7%)	C1 (8) C2 (41) C3 (25)	12	0
Lüdi et al., 2023 [12]	Combined Plating	Retrospective case series	34 (34)	57	10 (29.2%)	C1 (3) C2 (2) C3 (29)	48	23 (67%) Allograft
Kibar et al., 2021 [6]	Combined Plating	Retrospective case series	20 (20)	47	8 (40%)	C3 (20)	21	13 (65%) Allograft
Farhan et al., 2015 [7]	Combined Plating	Retrospective case series	24 (24)	53.3	-	C3 (24)	17	8 (33%) Autograft
Lundqvist et al., 2022 [18]	Combined Plating	Randomized controlled trial	74 (74)	62	17 (23%)	C1 (19) C2 (28) C3 (27)	12	-
Beeres et al., 2021 [1]	Bridge Plating	Retrospective case series	9 (9)	53.3	-	C1 (6) C2 (2) C3 (1)	3	-
Gruenberger et al., 2024 [10]	Bridge Plating	Retrospective cohort study	8 (8)	61.4	-	C2 (2) C3 (6)	2	Allografts
Lauder et al., 2015 [19]	Bridge Plating	Retrospective cohort study	18 (18)	61	13 (72.2%)	C3 (18)	32.4	-
Richard et al., 2012 [20]	Bridge Plating	Retrospective case series	33 (33)	70	10 (30.3%)	C2 or C3 (33)	11.8	0
Rambau et al., 2023 [21]	Bridge Plating	Retrospective case series	23 (23)	47.5	16 (68%)	C3 (23)	2.9	12 (52%) Allograft
Henry et al., 2021 [22]	Bridge Plating	Retrospective cohort study	60 (60)	65.5	15 (25%)	C2 (15) C3 (45)	10.2	-
Sharareh et al., 2019 [23]	Bridge Plating	Retrospective case series	24 (24)	41	14 (71%)	C3 (24)	4.9	-
Calem et al., 2025 [24]	Bridge Plating	Retrospective cohort study	35 (35)	43	24 (68.6%)	C3 (35)	5.8	0

**Table 2:** Functional and Radiographic Outcomes.

**Authors**	**VAS Scores (at Rest, with Activity)**	**Grip Strength (% Compared to Uninjured Side)**	**ROM (Ext-Flex)**	**DASH**	**PRWE**	**Batra Score**	**Volar Tilt (°)**	**Radial Height (mm)**
Medlock et al., 2018 [9]	-	71	42–36	30	35	-	9.1	-
Karlsson et al., 2020 [17]	0, 2	76	55–55	16	14	88	-	-
Lundqvist et al., 2022 [2]	0, 0	96	60–50	4.5	4.5	88	-	-
Sagerfors et al., 2020 [5]	0, 2	82	50–60	14.8	18	88	2.5	11
Lüdi et al., 2023 [12]	-	93	52–49	11	11	-	11	13
Kibar et al., 2021 [6]	2.1	78	52.2–48.7	10	-	-	8.6	10.5
Farhan et al., 2015 [7]	-	86	52–49	-	-	-	5	8.5
Lundqvist et al., 2022 [18]	0, 2	-	50–55	12.5	13.5	88	-	-
Beeres et al., 2021 [1]	-	-	-	32.9	-	-	-	-
Gruenberger et al., 2024 [10]	-	86	45–50	-	-	-	-	-
Lauder et al., 2015 [19]	-	-	46–43	16	14	-	-	-
Richard et al., 2012 [20]	-	-	50–46	32	-	-	-	-
Rambau et al., 2023 [21]	-	-	-	-	-	-	6.3	11.3
Henry et al., 2021 [22]	-	-	53.43–48.17	23.07	-	-	4.13	10.03
Sharareh et al., 2019 [23]	-	-	-	-	-	-	1.4	11.1
Calem et al., 2025 [24]	-	-	-	-	-	-	1.3	9.3

**Table 3:** Postoperative Complications.

Authors	Reoperation Rates (%) *	Hardware Removals (%)	Incidence of CRPS (%)	Infections (%)	Tendon Injuries (%)
Medlock et al., 2018 [9]	0	0	0	0	0
Karlsson et al., 2020 [17]	3 (3.85%) - 1 debridement for infection - 1 EIP to EPL tendon transfer and ECRB/ECRL repair with PL interposition graft - 1 FPL repair with PL interposition graft	18 (23.07%)	-	3 (3.85%)	2 (2.56%)
Lundqvist et al., 2022 [2]	3 (3.09%) - 2 debridements for infection - 1 corrective osteotomy	50 (51.55%)	0	2 (2.06%)	2 (2.06%)
Sagerfors et al., 2020 [5]	0	21 (28.37%)	0	2 (2.70%)	0
Lüdi et al., 2023 [12]	11 (32.35%) - 1 revision fixation for loss of reduction - 3 scar revisions - 1 intra-articular screw removal - 1 EDC repair - 1 dorsal joint capsule repair for carpal instability - 3 corrective osteotomies - 1 ulnar styloid non-union resection and wafer procedure	32 (94.12%)	3 (8.82%)	2 (5.88%)	1 (2.94%)
Kibar et al., 2021 [6]	0	0	2 (10.00%)	0	0
Farhan et al., 2015 [7]	0	4 (16.67%)	0	0	0
Lundqvist et al., 2022 [18]	1 (1.35%) - 1 debridement for infection	23 (31.08%)	0	3 (4.05%)	0
Beeres et al., 2021 [1]	3 (33.33%) - 1 extensor tenolysis - 1 peri-implant fracture open reduction internal fixation - 1 EIP to EPL tendon transfer	9 (100%)	1 (11.11%)	1 (11.11%)	1 (11.11%)
Gruenberger et al., 2024 [10]	1 (12.5%) - 1 carpal tunnel release and flexor tenolysis	8 (100%)	0	0	0
Lauder et al., 2015 [19]	0	18 (100%)	0	0	0
Richard et al., 2012 [20]	1 (3.03%) - 1 debridement for infection	33 (100%)	1 (3.03%)	1 (3.03%)	0
Rambau et al., 2023 [21]	0	23 (100%)	0	0	0
Henry et al., 2021 [22]	2 (3.33%) - 1 flexor tenolysis - 1 EIP to EPL tendon transfer	60 (100%)	1 (1.67%)	4 (6.67%)	2 (3.33%)
Sharareh et al., 2019 [23]	3 (12.5%) - 1 extensor tenolysis - 1 carpal tunnel release - 1 broken plate removal	24 (100%)	-	0	0
Calem et al., 2025 [24]	2 (5.71%) - 1 broken plate removal - 1 broken plate removal with cancellous allograft insertion for partial union	35 (100%)	-	0	0

* Excluding hardware removals for intact hardware.

## Data Availability

All extracted data are available within the tables included in the article.

## Supplementary Materials

The following supporting information can be downloaded at: https://www.mdpi.com/article/10.3390/jcm15155991/s1, Table S1: PRISMA Checklist.

## Abbreviations

The following abbreviations are used in this manuscript:

EF	External fixation
VLP	Volar locking plate
ROM	Range of motion
PRISMA	Preferred Reporting Items for Systematic reviews and Meta-Analyses
MINORS	Methodological Index for Non-Randomized Studies
GRADE	Grading of Recommendations Assessment, Development, and Evaluation
RCT	Randomized controlled trial
DASH	Disabilities of the Arm, Shoulder, and Hand
PRWE	Patient-Rated Wrist Evaluation
CRPS	Complex regional pain syndrome
EIP	Extensor indicis propius
FPL	Flexor pollicis longus
EPL	Extensor pollicis longus
DBP	Dorsal bridge plating
DRUJ	Distal radioulnar joint

## Appendix A. Search Strategy

(“Dual plat*” OR “Combined plat*” OR “Volar-dorsal plat*” OR “Dorsal-volar plat*” OR “Double plat*” OR “Dual fixation” OR “Volar-dorsal fixation” OR “Dorsal-volar fixation” OR “Double fixation” OR “Fragment-specific fixation”) OR (“Bridge plat*” OR “Spanning plat*” OR “Distraction plat*” OR “Internal external fixator” OR “Bridge fixation” OR “Spanning fixation” OR “Distraction fixation”) AND (“distal radius fractur*” OR “Distal radius injur*” OR “Radius fractur*” OR “Radius injur*” OR “Radius osteosynthesis” OR “Radius ORIF” OR “Radius open reduction internal fixation”)

No limits or extra filters were used.
